# Effect of Posterior Cruciate Ligament Resection on Gap Balancing in Robot‐assisted Total Knee Arthroplasty

**DOI:** 10.1111/os.14135

**Published:** 2024-06-11

**Authors:** Kechao Zhu, Jiaxing Wang, Huiyong Dai, Yan Xi, Qiaojie Wang, Xianlong Zhang, Qi Wang

**Affiliations:** ^1^ Department of Orthopedics Shanghai Sixth People's Hospital Affiliated to Shanghai Jiao Tong University School of Medicine Shanghai China; ^2^ Shanghai First‐Imaging Information Technology Co., Ltd Shanghai China

**Keywords:** Bone resection, Femoral component rotation, Flexion–extension gap, Posterior cruciate ligament, Total knee arthroplasty

## Abstract

**Objective:**

Retention or sacrifice of the posterior cruciate ligament (PCL) is one of the most controversial issues while performing total knee arthroplasty (TKA). This study aimed to evaluate the impact of PCL resection on flexion–extension gaps, femoral component rotation, and bone resection amounts during robot‐assisted TKA.

**Methods:**

This prospective study included 40 patients with knee osteoarthritis who underwent robot‐assisted posterior‐stabilized (PS) TKA between September 2021 and February 2022. Of the patients, 75% were women (30/40) with a mean age and BMI of 72.6 years and 27.4 kg/m^2^, respectively. The guidance module and camera stand assembly were used to capture gaps before and after PCL resection. Measurements of femoral component rotation and bone resection amounts were made in cruciate‐retaining (CR) TKA mode and PS‐TKA mode.

**Results:**

After PCL resection, the mean change in the medial and lateral compartments of flexion gaps increased by 2.0 and 0.6 mm, respectively (*p* < 0.001). Compared with the CR‐TKA mode group, the bone resection amounts of the medial posterior condyle and the lateral posterior condyle in the PS‐TKA mode group decreased by 2.0 ± 1.1 and 1.1 ± 1.1 mm, respectively, and the external rotation of the femoral prosthesis relative to the posterior condylar axis and trans‐epicondylar line was reduced by 1.0° ± 1.3° and 1.2° ± 1.6°, respectively (*p* < 0.001).

**Conclusion:**

The release of the PCL did not affect the extension gap, but significantly increased the flexion gap. Moreover, the increases in the medial flexion gap were greater than those of the lateral flexion gap. After PCL resection, less external rotation of the femoral prosthesis and fewer bone cuts of the posterior femur were needed in PS‐TKA.

## Graphical abstract

Flowchart showing the study design of the clinical trial.

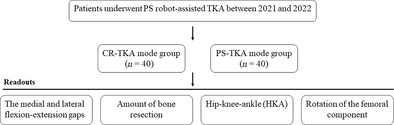

## Introduction

Total knee arthroplasty (TKA) is considered an effective treatment for end‐stage osteoarthritis of the knee.^1^ Despite this, 15%–30% of patients are still dissatisfied, or not completely satisfied, with the clinical outcomes of TKA.^2^, ^3^ Soft‐tissue imbalance, instability, malalignment, and rotational malalignment of the component are major factors that affect functional outcomes and long‐term implant survival following TKA.^4^, ^5^, ^6^ In the knee joint, four major ligaments, the anterior cruciate ligament (ACL), the posterior cruciate ligament (PCL), the lateral collateral ligament (LCL), and the medial collateral ligament (MCL), altogether play very important roles in knee stability and kinematics. Inadequate correction of soft tissue imbalances, unbalanced flexion–extension gaps, and knee instability after primary TKA are major contributing factors to early TKA failures.^7^, ^8^ While the LCL and MCL are kept intact to maintain joint stability in knee arthroplasty, the ACL is usually removed regardless of whether it is a posterior‐stabilized (PS) or cruciate‐retaining (CR) knee prosthesis. The PCL, as the strongest ligament in the knee joint, plays a critical role in restricting posterior tibial displacement and stabilizing the knee.^9^ A debate has raged for many years concerning whether the PCL should be preserved or resected during TKA, and research has also focused on exploring the impact of PCL resection on flexion–extension gaps.^10^, ^11^ Historical studies performed on cadaveric specimens reported that the main effect of PCL sacrifice is a larger flexion gap, with an increase of approximately 4 mm or more.^12^, ^13^ However, cadaveric knees cannot reflect the physiological state of a normal knee joint. Few studies have reported that PCL resection increases both flexion and extension gaps.^14^, ^15^ In patients with severe osteoarthritis, the collateral ligament and cruciate ligament may need to bear an extra load, which may result in a difference in flexion gaps after PCL resection.

Accurate bone resection and soft tissue balancing are of great importance in correcting deformity, equalizing the flexion and extension gaps, and restoring stability.^16^ Therefore, changes in flexion–extension gaps following PCL resection may lead to alterations in bone resection, femoral rotation, and the size of the femoral component during TKA to obtain a balanced knee joint.^11^ Previous studies have mostly focused on the changes to flexion–extension space after PCL resection,^14^, ^17^ yet it is challenging to evaluate the contribution of PCL resection to the amount of osteotomy and femoral rotation during traditional TKA surgery. In the present study, gap and bone resection amounts and femoral component rotation could be accurately evaluated before real bone cut by utilizing the robot‐assisted TKA system. Therefore, the surgeon is able to detect changes in gap distance, femoral rotation, and bone resection amounts before and after PCL resection.

Considering that there is no consensus on the effect of PCL sacrifice on the biomechanics of the knee, a better understanding of the role of the PCL in TKA may contribute to improving its applicability in current practices. The primary objective of this study was to analyze the effect of PCL resection on intraoperative flexion–extension gaps. The hypothesis was that PCL resection would increase the flexion gap more than the extension gap, and that the medial and lateral flexion gaps would increase unevenly. The second hypothesis of this study was that less bone resection and femoral component rotation were needed following PCL resection.

## Methods

### Study Design

This prospective study was registered as a clinical trial (No. ChiCTR2100049679). Institutional review board approval (No.2021‐185) was received to review this prospectively collected data on 40 TKAs. All patients aged 18–85 years, undergoing PS robot‐assisted TKA performed by a senior surgeon at our institution between September 2021 and February 2022 owing to varus osteoarthritis of the knee joint were retrospectively reviewed and included in this study. The Triathlon (Stryker; Kalamazoo, MI, USA) posterior‐stabilized (PS) prosthesis was implanted with the MAKO robot‐assisted system (Stryker). Patients with disabling knee pain and a radiographic diagnosis of varus primary end‐stage knee osteoarthritis were recruited. Exclusion criteria were osteoarthritis after pathologic or traumatic fractures, valgus osteoarthritis, rheumatoid arthritis, patients with an incompetent PCL, and a flexion contracture > 25°.

Using the method of intraoperative simulation, the CR‐TKA mode group and PS‐TKA mode group were established. That is, for the patients who underwent robot‐assisted PS‐TKA, osteophyte removal and ligament release, if necessary, after joint exposure, gap balancing was performed in CR‐TKA mode with the MAKO robot system before resecting the PCL. After resecting the PCL, gap balancing was re‐performed in the PS‐TKA mode. The medial and lateral flexion–extension gaps, amount of bone resection, hip‐knee‐ankle (HKA) angle, and rotation of the femoral component in both modes were recorded.

### Surgical Technique

All surgeries were performed by a senior surgeon, and a conventional medial parapatellar approach was used in all cases. Tracker arrays were secured on the femoral and tibial shafts, and two checkpoints were placed on the femur and tibia to ensure that the trackers had not moved before resection. The femoral and tibial bones were registered using a sharp probe. After registration, the degree of varus deformity, maximum knee extension and flexion, angle of flexion contracture, and flexion–extension gaps were evaluated and recorded. Osteophytes of the distal femur and medial tibial plateau were removed without the release of soft tissue, and the flexion–extension gaps were assessed before ACL excision. The ACL was then resected and the integrity of the PCL was evaluated by the surgeon. The intraoperative findings of PCL deficiency were not included in this study.

Extension and flexion gaps were evaluated with the surgeon holding the distal tibia with one hand and exerting varus or valgus stress at the level of the tibial tuberosity with the other hand. The assistant avoided rotation of the femur by holding the proximal femur in both hands. With the robot‐assisted system, limb alignment was visualized, and flexion and extension gaps were identified. During the procedure, the screen recordings of measurements were performed by the assistant, while the surgeon did not observe the visual data on the screen at the same time. The mean value was calculated for the medial and lateral gaps after three measurements were obtained.

**FIGURE 1:**
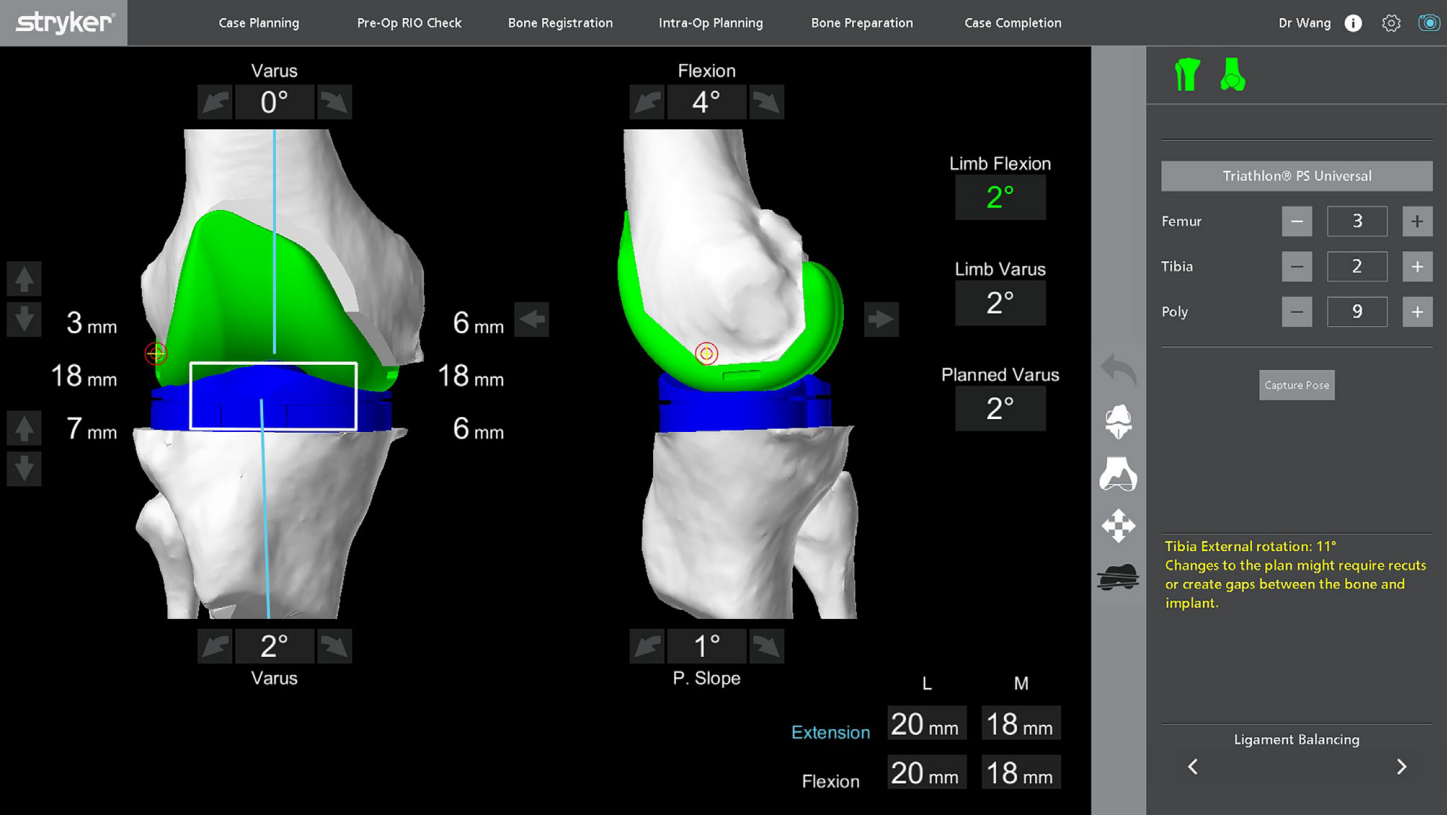
Ligament balancing. The image illustrates the balanced flexion–extension gaps after ligament balancing during TKA surgery. TKA, total knee arthroplasty.

Gap balancing was achieved by fine‐tuning the position of the implant and always started with a 2° varus of the tibial plateau if more medial gap opening was needed, followed by varus adjustment of the femoral component in extension and external rotation of the femoral component in flexion until the flexion and extension gaps were balanced (Figure 1). During intraoperative data recording, the posterior tibial slope was set at 3° for both CR‐TKA and PS‐TKA modes, and the lateral plateau resection thickness was set at 7 mm. The maximum residual varus allowed after fine‐tuning of the component was 5°, and medial release would be needed if gap balancing was not achieved within 5° of varus.

According to the MAKO system manual, the ideal target flexion–extension gap is 18 mm. However, owing to the large individual differences between patients, for example, in patients with obvious lateral laxity, an equal medial and lateral gap of 18 mm is not always achievable. Based on the surgeons' experience, we established a protocol to adjust the flexion–extension gap: (i) if the gap difference between the medial and lateral sides was <2 mm, 18 mm of medial gap and lateral gap were established; (ii) if the gap difference between the medial and lateral sides was 2–4 mm, 18 mm of medial gap and 19 mm of lateral gap were established; and (iii) if the gap difference between the medial and lateral sides was >4 mm, 18 mm of medial gap and 20 mm of lateral gap were established.

**FIGURE 2:**
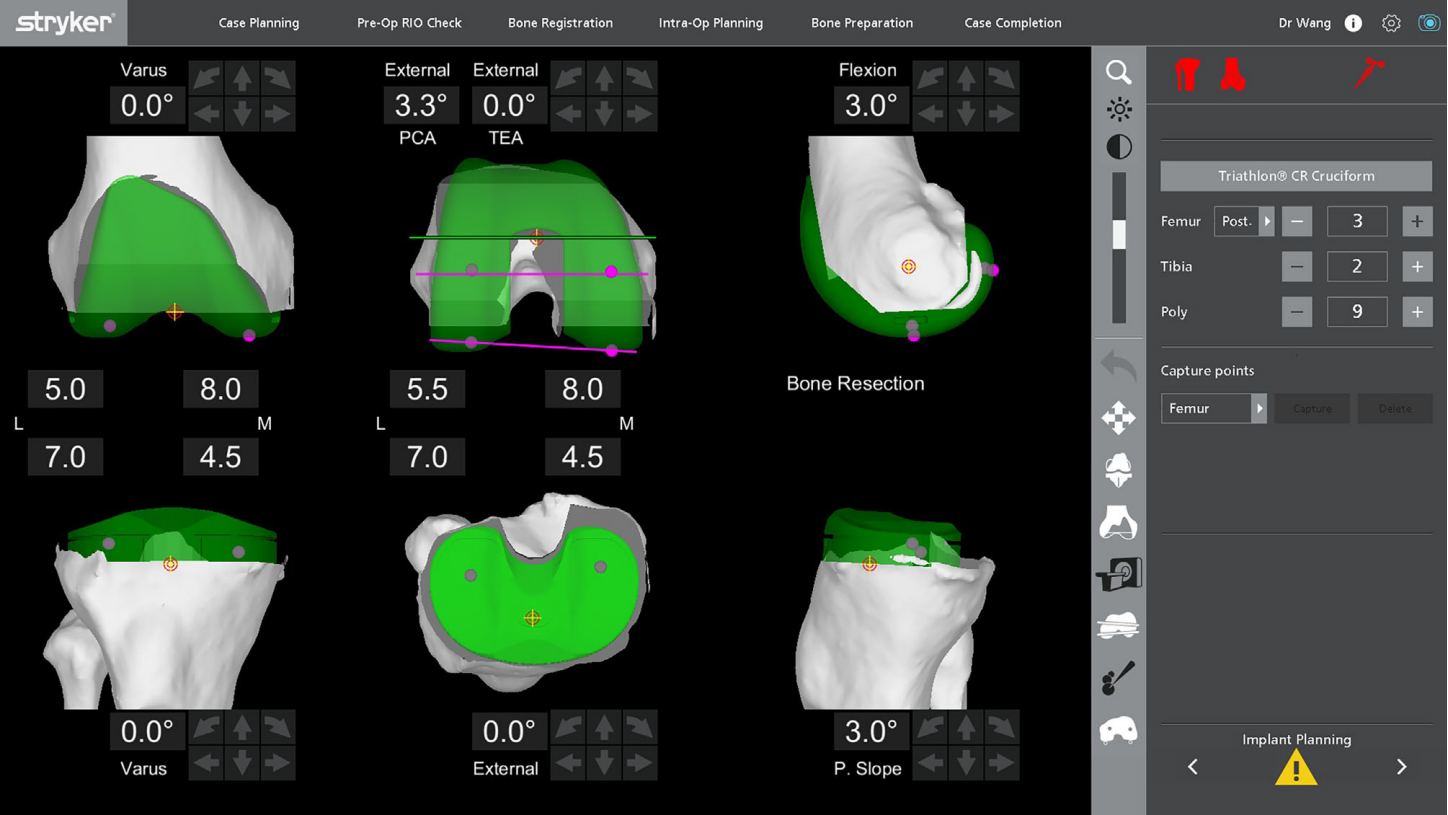
Intraoperative screenshot of component positions. The image illustrates the thickness of the bone cut and rotation of the femoral component.

The screen of the robot‐assisted system showed the fine‐tuning parameters of the implant, planned amount of osteotomy, HKA, and rotation of the femur component (Figure 2). The excision thickness of the femur and tibia did not consider the residual cartilage. In this study, the rotation compared to the posterior condylar axis and trans‐epicondylar line were defined as PCA and TEA, respectively. Positive values corresponded to varus alignment and external rotation.

No bone resection was performed in the CR‐TKA mode, and the implant position was restored to the original plan. The PCL was then resected from its femoral insertion and tibial plateau using electrocautery until the surgeon confirmed that the PCL had been completely removed. Next, the medial and lateral gaps in extension and flexion were re‐evaluated in the PS‐TKA mode, and gap balancing was re‐performed using the same techniques described above. After recording all the measurements, the posterior tibial slope was then adjusted to 1°, and the gap balancing was re‐performed. Following bone resection, trial prosthetic components were implanted, the alignment and gaps were checked, and the final prosthesis was implanted.

### Statistical Analysis

At 80% power with an alpha of 0.05, a sample size of at least 34 patients was required to evaluate a 1.5 mm change in medial flexion gaps after PCL resection. SPSS (Version 25, IBM Corporation; Armonk, NY, USA) was used to analyze the data. The normal distribution of measurement data is expressed as ($\bar{x}$ ± s). The Shapiro–Wilk test was used to test for the normal distribution of continuous variables, and boxplots were used for visual confirmation. Non‐paired and paired continuous data from the two groups were compared using Student's two‐sample *t*‐test and a paired two‐sample *t*‐test, respectively. Correlations between continuous variables were evaluated using Pearson's (*r*) coefficient. Based on the three gap measurements recorded for each outcome, the intraclass correlation coefficients (ICCs) were calculated. For all statistical tests, a *p* value < 0.05 was considered significant.

## Results

The 40 enrolled patients had a preoperative varus deformity and underwent PS‐TKA using the MAKO robot system. All demographic, preoperative, and postoperative data of the patients are listed in Table 1.

**TABLE 1:** Demographics and characteristics of the patients.

Variable	Outcome
Number	40
Age (years)	72.6 ± 4.5 (59 to 82)
Sex	
Male	10/40 (25.0%)
Female	30/40 (75.0%)
BMI (kg/m^2^)	27.4 ± 3.5 (20.7 to 37.5)
Preoperative HKA, varus (degree)	8.5° ± 4.8° (0° to 22°)
Range of motion (degree)	131.9° ± 6.4° (115° to 145°)
Preoperative flexion contraction (degree)	7.9° ± 6.3° (−3° to 23°)
Postoperative HKA, varus (degree)	2.9° ± 1.3° (0° to 5°)
Postoperative flexion contraction (degree)	5.0° ± 2.6° (−1° to 11°)

*Notes*: A positive value refers to varus alignment. The outcomes are presented as Mean ± SD (range).

Abbreviations: HKA, hip‐knee‐ankle; BMI, body mass index.

### Gap Measurements

**FIGURE 3:**
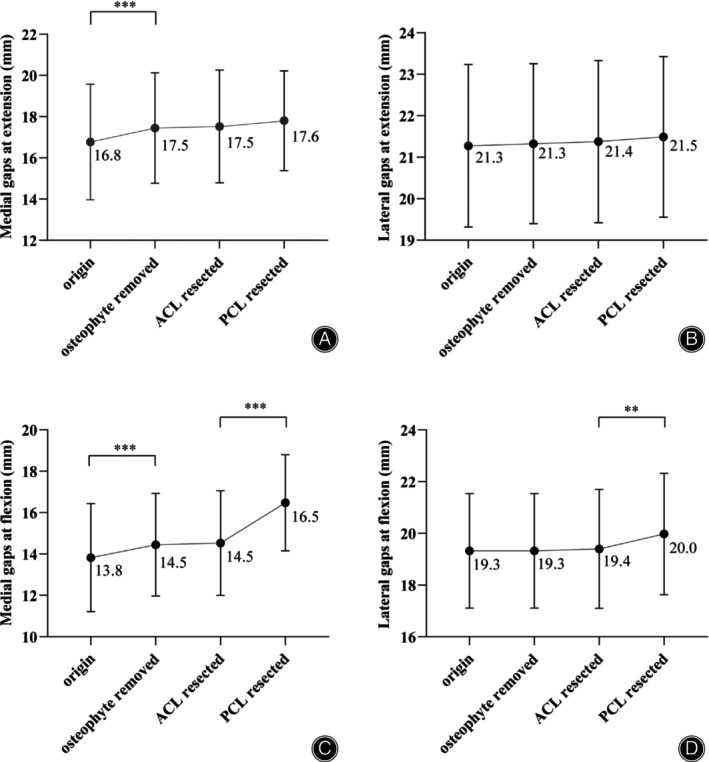
Measurement of joint space in origin state, after osteophyte removal, after ACL resection, and after PCL resection. (A) medial gaps at extension. (B) lateral gaps at extension. (C) medial gaps at flexion. (D) lateral gaps at flexion. (** represents *p* < 0.01, *** represents *p* < 0.001). ACL, anterior cruciate ligament; PCL, posterior cruciate ligament.

Both the medial and lateral gaps were measured in millimeters at extension (10°–20° degree flexion) and 85°–95°degree of flexion. The medial and lateral compartments of the flexion–extension gap dimensions are reported in Table 2. After the osteophyte was removed, the medial joint gaps increased by 0.7 mm at extension and 0.7 mm at flexion (Table 2, Figure 3, *p* < 0.001). However, no significant difference was noted in the flexion and extension gaps in either the medial or lateral compartment after ACL resection. The average flexion joint space dimension progressively and significantly increased after PCL resection by 2.0 mm (*p* < 0.001) at the medial and 0.6 mm (*p* < 0.01) at the lateral (Table 2, Figure 3) sides. After ACL resection and before the PCL resection, the extension gap in the medial and lateral were 17.5 ± 2.7 and 21.4 ± 1.9 mm, respectively. With PCL resection, the extension gap in the medial and lateral were 17.6 ± 2.4 and 21.5 ± 2.0 mm, respectively (Table 2, Figure 3). Following PCL resection, there was no significant difference in medial gaps (0.1 mm; *p* = 0.06) and lateral (0.1 mm; *p* = 0.08) in extension.

**TABLE 2:** Comparison of joint gaps in extension and flexion.

Measure	Compartment	Origin (mm) (SD)	Osteophyte removed (mm) (SD)	ACL resected (mm) (SD)	PCL resected (mm) (SD)
Extension gap	Medial	16.8 (2.8)	17.5 (2.7)	17.5 (2.7)	17.6 (2.4)
	Lateral	21.3 (1.9)	21.3 (1.9)	21.4 (1.9)	21.5 (2.0)
Flexion gap	Medial	13.8 (2.6)	14.5 (2.5)	14.5 (2.5)	16.5 (2.3)
	Lateral	19.3 (2.2)	19.3 (2.2)	19.4 (2.3)	20.0 (2.3)

Abbreviations: ACL, anterior cruciate ligament; PCL, posterior cruciate ligament.

**FIGURE 4:**
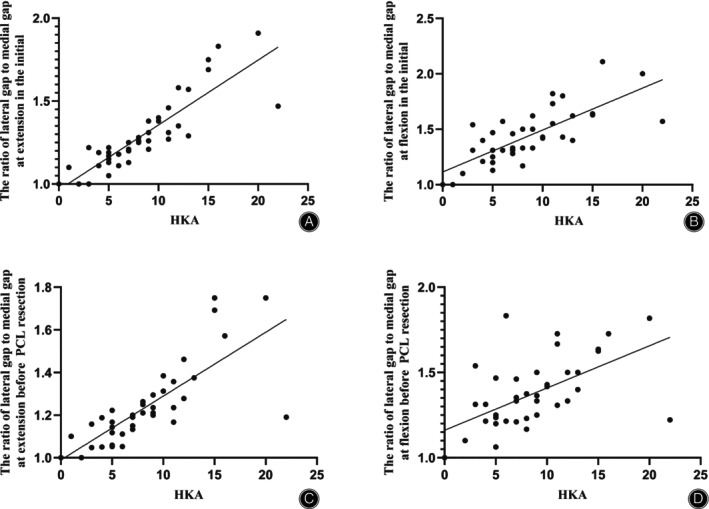
The correlation between the HKA and flexion–extension gaps. (A) The extension gaps in the initial. (B) The flexion gaps in the initial. (C) The extension gaps before PCL resection. (D) The flexion gaps before PCL resection. HKA, hip‐knee‐ankle; PCL, posterior cruciate ligament.

In addition, the mean ratio of the origin lateral gap to the medial gap was 1.3 ± 0.2 at extension and 1.4 ± 0.2 at flexion, respectively. After osteophytes were removed and the ACL was resected, the mean ratio of the lateral gap to the medial gap was 1.2 ± 0.2 at extension and 1.4 ± 0.2 at flexion, respectively. The degree of varus was positively correlated with the ratio of the lateral to the medial gaps. The correlation coefficients were 0.9 at extension and 0.8 at flexion, respectively (*p* < 0.01) (Figure 4). After removing the osteophytes and cutting the ACL, the correlation coefficients were 0.8 at extension and 0.6 at flexion, respectively (*p* < 0.01) (Figure 4).

### Bone Resection Amount Measurements

**FIGURE 5:**
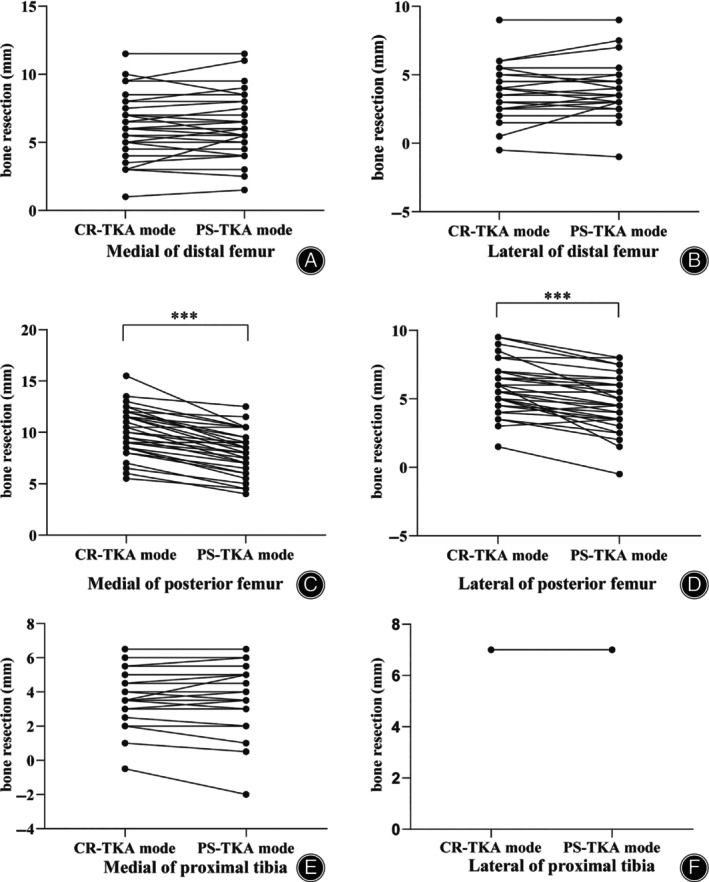
Resection levels between CR‐TKA and PS‐TKA. (A) Medial of the distal femur. (B) Lateral of the distal femur. (C) Medial of the posterior femur. (D) Lateral of the posterior femur. (E) Medial of the proximal tibia. (F) Lateral of the proximal tibia. (*** represents *p* < 0.001). CR‐TKA, cruciate retaining total knee arthroplasty; PS‐TKA, posterior stabilized total knee arthroplasty.

After gap balancing, the number of bone resections of the femur and tibia was collected without real bone cuts from the MAKO robot system, which were displayed on the screen (Table 3). The mean posterior femoral bone cut amount was decreased to 8.1 mm on the medial side and 4.7 mm on the lateral side in the PS‐TKA mode, compared with 10.1 mm on the medial side and 5.8 mm on the lateral side in CR‐TKA mode (Table 3, Figure 5, *p <* 0.001). With respect to the amount of bone resection of the distal femur and proximal tibia, the change did not differ on the medial or lateral sides.

**TABLE 3:** Comparison of bone resection amounts.

Measure	Compartment	Origin (mm) (SD)	CR‐TKA mode (mm) (SD)	PS‐TKA mode (mm) (SD)
Femur distal	Medial	7.8 (0.6)	6.3 (2.2)	6.4 (2.2)
Femur distal	Lateral	5.5 (2.0)	3.4 (1.8)	3.5 (1.8)
Femur posterior	Medial	8.0 (0.2)	10.1 (2.2)	8.1 (2.1)
Femur posterior	Lateral	5.2 (1.7)	5.8 (1.8)	4.7 (1.9)
Tibia proximal	Medial	2.4 (1.8)	3.6 (1.5)	3.6 (1.8)
Tibia proximal	Lateral	7.0 (0.0)	7.0 (0.0)	7.0 (0.0)

Abbreviations: CR‐TKA, cruciate retaining total knee arthroplasty; PS‐TKA, posterior stabilized total knee arthroplasty.

### Limb Alignment and Femoral Component Rotation

**FIGURE 6:**
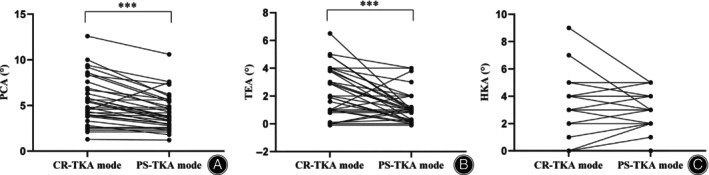
Femoral prosthesis rotation angle and HKA between CR‐TKA and PS‐TKA. (A) PCA. (B) TEA. (C) HKA. (*** represents *p* < 0.001). CR‐TKA, cruciate retaining total knee arthroplasty; PS‐TKA, posterior stabilized total knee arthroplasty; TEA, trans‐epicondylar line; PCA, posterior condylar axis.

The rotation of the femoral component was evaluated after gap balancing. Table 4 presents intraoperative information on limb alignment and femoral rotation. The mean PCA in CR‐TKA mode was 5.5° ± 2.5° (range, 1.3° to 12.6°), and the mean TEA was 2.3° ± 1.6° (range, 0° to 6.5°). The mean PCA in PS‐TKA mode was 4.5° ± 2.1° (range, 1.2° to 10.6°), and the mean TEA was 1.1° ± 1.1° (range, 0° to 3.8°). In comparison with the CR‐TKA group, lesser external rotation of the femoral component was needed in the PS‐TKA group; it decreased by 1.0° for PCA and 1.2° for TEA, respectively (Table 4, Figure 6, *p <* 0.001).

**TABLE 4:** A comparison of femoral prosthesis rotation and Hip‐Knee‐Ankle Angle (HKA).

Measure	Origin (°) (SD)	CR‐TKA mode (°) (SD)	PS‐TKA mode (°) (SD)
PCA	3.2 (1.9)	5.5 (2.5)	4.5 (2.1)
TEA	0.0 (0.0)	2.3 (1.6)	1.1 (1.1)
HKA	8.5 (4.8)	2.8 (1.8)	2.7 (1.3)

Abbreviations: CR‐TKA, cruciate retaining total knee arthroplasty; HKA, hip‐knee‐ankle; PCA, posterior condylar axis; PS‐TKA, posterior stabilized total knee arthroplasty; TEA, trans‐epicondylar line.

The HKA in the CR‐TKA group and PS‐TKA group were 2.8° ± 1.8° and 2.7° ± 1.3°, respectively (Table 4, Figure 6). No difference was observed in HKA between the two groups. Moreover, no correlations were observed between changes in the origin of the HKA and CR‐TKA or PS‐TKA.

## Discussion

Our principle finding supports the hypothesis that PCL resection results in a larger increment in the flexion gap as compared to the extension gap. PCL resection increased the medial flexion gap more than the lateral flexion gap. Less bone resection and femoral component rotation were needed following PCL resection. Limb alignment remained unaffected by PCL resection.

### Flexion and Extension Gaps

A well‐balanced flexion–extension gap and correct alignment of the components are the primary goals of orthopedic surgeons while performing TKA.^18^ Our study found a notable disparity in the impact of PCL resection on the flexion and extension gaps during TKA, with a considerably greater increase observed in the flexion gap. According to the literature, PCL resection has a greater effect on the opening of the flexion gap, potentially causing a flexion–extension mismatch.^13^, ^14^, ^15^, ^17^, ^19^, ^20^ Kadoya *et al*.^14^ analyzed 30 patients with PCL resection in TKA and found that the flexion gap significantly increased on the medial and the lateral sides (4.8 and 4.5 mm, respectively), but that there was no effect on the extension gap. Warth *et al*.^17^ reviewed the outcomes of 129 standardized TKAs and reported that the tibiofemoral gap increased progressively from extension to flexion after PCL resection. Tu *et al*.^19^ reported that in 33 patients with knee osteoarthritis, the major result of PCL release was the creation of a larger flexion gap. As demonstrated in our study, ACL resection did not affect both the extension and flexion gaps, while the release of the PCL significantly increased the flexion gap, but did not have any measurable effect on the extension gap.

In accordance with some studies,^11^, ^20^, ^21^ another finding of the current study is that sacrificing the PCL increased the medial flexion gap more than the lateral flexion gap. Schnurr *et al*.^20^ studied 50 patients and found a 1.3 mm medial and 0.7 mm lateral increase in the 90° flexion gap after PCL resection. Chaiyakit *et al*.^21^ showed that PCL resection leads to a larger flexion gap, with the medial gap increasing by 2.0 mm and the lateral gap increasing by 1.6 mm, which are similar to the values in flexion in our study. Park *et al*.^11^ reviewed the outcomes of 30 osteoarthritis patients with severe varus deformity or flexion contracture and found that the medial and lateral flexion gaps increased significantly, by 4.5 and 3.4 mm, respectively. Park *et al*. used a tensioning device with a larger distraction force (200 Nm) to measure gaps and performed medial release to correct the varus deformity before PCL resection, which might have increased the gap measurements. In this study, we did not perform a medial soft tissue release unless the gap between the medial and lateral sides was too large, which might cause difficulties in ligament balance.

Other studies have indicated that PCL resection increases the lateral flexion gap more than the medial flexion gap.^15^, ^22^ Some researchers have reported that varus knee deformity results in a progressive laxity of the lateral soft tissues.^23^, ^24^ Our results showed that the greater the varus deformity, the greater the ratio of the lateral to medial gaps, which indicates that varus deformity is correlated with an imbalance of the medial and lateral gaps. Even after the osteophytes were removed and the ACL resected, that is, before PCL resection, a correlation still exists. In Kayani *et al*.,^15^ the mean preoperative varus deformity was smaller than that reported in our study 4.1° (SD 3.4°) versus 8.5°(SD4.8°). Patients with a severe varus deformity may have a larger lateral gap, which may affect the lateral gap after PCL resection. The current study found that the increase in the medial flexion gap was larger than that of the lateral gap after PCL resection, which may create medial laxity and result in less external rotation of the femoral component. Moreover, it may be easier to obtain a balanced gap in patients with severe varus deformity, especially the balance of the medial and lateral flexion gaps.

### Femoral Component Rotation

Studies on the influence of PCL resection on femoral component rotation are scarce. Compared to pre‐ and post‐PCL resection in femoral component planning, the mean external rotation of the femoral component decreased by 1.2° (from 2.3° to 1.1°) in the current study. These findings are consistent with those of Park *et al*.,^11^ who reported that the mean external rotation of the femoral component decreased by 1.6° after PCL resection. It is generally believed that varying degrees of femoral component rotation may influence the biomechanical knee function.^25^ In traditional TKA, the femoral component is rotationally aligned parallel to the trans‐epicondylar axis to optimize the patellofemoral and tibiofemoral kinematics.^26^ Our results indicate that the PCL‐sacrificed design may lead to an external rotation of the femoral component closer to the trans‐epicondylar line. In some patients who require external rotation to obtain balanced gaps during TKA, the PS prosthesis may be a more suitable choice.

### Bone Resection

To the best of our knowledge, few studies have evaluated the amount of bone resection before and after PCL resection. Using the MAKO robot system, gap balancing was performed, and measurements of bone resection were recorded in both CR‐TKA and PS‐TKA modes. In patients with a severe flexion contracture, soft tissue balancing and additional distal femur bone cutting were recommended during TKA to correct this.^27^, ^28^ However, in our group of patients, we did not perform additional distal femur bone cutting. On the contrary, the resection amount of the distal femur was decreased compared with the preoperative plan, regardless of the use of the CR or PS prosthesis. Randomly increasing the amount of distal femoral osteotomy may result in extension instability. Although a lesser amount of distal femur bone was resected, our study showed that the mean preoperative flexion contracture was 7.9° ± 6.3°, and was corrected to 5.0° ± 2.6° after prosthesis implantation.

Our study showed that the mean posterior femoral bone cut amount was decreased by 2.0 mm (from 10.1 to 8.1 mm) at the medial and 1.1 mm (from 5.8 to 4.7 mm) at the lateral after PCL resection. Vermue *et al*.^29^ analyzed 268 patients who underwent robot‐assisted TKA and found that removing the same amount of bone from the distal and posterior femur resulted in an imbalance in the flexion–extension gap. Excessive external rotation and posterior condyle cuts may decrease the medial posterior condyle offset, which may affect maximal knee flexion.^30^, ^31^ Our findings indicate that more external rotation of the femoral component and more medial posterior femur bone resection may be needed in CR‐TKA. By switching from a CR to PS prosthesis, less bone cutting may be required, and more bone mass might be preserved for these patients.

### Strengths and Limitations

This study was based on virtual robot‐assisted surgery and not a real postoperative comparative study. There are several limitations to consider. First, the sample size was relatively small, although the number of samples was evaluated before the study to attain reasonable statistical power. Second, in this study, our gap measurements did not utilize devices such as tensioners. As mentioned above, there is still no consensus on how much tension should be applied to the device, and from literature reports, the force could vary from 100 to 200 Nm.^11^, ^19^, ^20^, ^32^ In our study, all measurements were manually performed by a single experienced surgeon, who determined the forces of the valgus/varus strains. The magnitude of valgus/varus stress is the force routinely used in daily surgeries to evaluate knee stability, and all assessments were repeated three times. It has been proven effective to use this technique for assessing soft‐tissue laxity in robot‐assisted knee arthroplasty.^33^

To our knowledge, there is much research regarding the effects of PCL resection in TKA. Most studies have focused on the impact of PCL resection on the flexion–extension gaps, while there is limited research regarding the effects of PCL resection on femoral component rotation and the amount of bone resection during the surgery. In this study, leveraging the advantages of real‐time feedback provided by the MAKO robot systems, we conducted gap balancing and intraoperative planning to evaluate the effects of PCL resection on the flexion–extension gap, femoral component rotation, and amount of bone resection in robot‐assisted TKA. Our results showed that varus deformity is associated with an imbalance between the medial and lateral gaps. The changes in flexion–extension gaps caused by PCL resection can affect the necessary rotation of the femoral component to achieve gap balance. This has clinical significance for surgeons in selecting the prosthesis and achieving lower limb alignment and soft tissue balance in TKA.

## Conclusion

In conclusion, when PS‐TKA is conducted, PCL resection results in a flexion–extension mismatch that causes the increase in the flexion gap to be greater than that of the extension gap. Moreover, the increase in the medial flexion space was significantly larger than that of the lateral flexion gap. Following PCL resection, less external rotation of the femoral component was required to obtain a balanced flexion/extension gap. Not all patients require additional distal femur bone to correct the flexion contracture. Less bone cutting of the posterior femur is needed in PS‐TKA, which may contribute to the preservation of bone mass. These findings can potentially provide valuable information for surgeons that routinely perform TKA to achieve a stable knee.

## Conflict of Interest Statement

No conflict of interest exists in the submission of this manuscript.

## Ethical Statement

All patients provided written informed consent. The study design was approved by research ethics committee of the Sixth People's Hospital affiliated with the Shanghai Jiao Tong University School of Medicine.

## Author Contributions

Conceptualization: Qi Wang and Xianlong Zhang. Data curation and formal analysis: Kechao Zhu and Jiaxing Wang. Investigation: Kechao Zhu and Huiyong Dai. Software and Methodology: Huiyong Dai and Yan Xi. Writing—review and editing: Kechao Zhu, Jiaxing Wang, Qiaojie Wang, Xianlong Zhang and Qi Wang. All authors have reviewed and approved the final version of the manuscript.

## Funding Information

This work was supported by The Excellent Youth Training Program of Shanghai Sixth People's Hospital Affiliated to Shanghai Jiao Tong University School of Medicine (Grant No. ynyq202202).

## Authorship Declaration

All authors listed meet the authorship criteria according to the latest guidelines of the International Committee of Medical Journal Editors, and all authors are in agreement with the manuscript.
